# Opportunities and Challenges for Including Oyster-Mediated Denitrification in Nitrogen Management Plans

**DOI:** 10.1007/s12237-021-00936-z

**Published:** 2021-12

**Authors:** Julie M. Rose, J. Stephen Gosnell, Suzanne Bricker, Mark J. Brush, Allison Colden, Lora Harris, Eric Karplus, Alix Laferriere, Nathaniel H. Merrill, Tammy B. Murphy, Joshua Reitsma, Johnny Shockley, Kurt Stephenson, Seth Theuerkauf, Dan Ward, Robinson W. Fulweiler

**Affiliations:** 1 NOAA Fisheries, NEFSC Milford Laboratory, 212 Rogers Ave, Milford, CT 06460, USA; 2 Department of Natural Sciences, Baruch College and the PhD Program in Biology, The Graduate Center of the City University of New York, 17 Lexington Avenue, New York, NY 10010, USA; 3 NOAA NCCOS Oxford Laboratory, 904 South Morris Street, Oxford, MD 21654, USA; 4 Virginia Institute of Marine Science, William & Mary, 1370 Greate Road, Gloucester Point, VA 23062, USA; 5 Chesapeake Bay Foundation, 6 Herndon Avenue, Annapolis, MD 21403, USA; 6 University of Maryland Center for Environmental Science, 146 Williams Street, Solomons, MD 20688, USA; 7 Science Wares, Inc., 87 Hamlin Ave, Falmouth, MA 02540, USA; 8 The Nature Conservancy, New Hampshire Chapter, 112 Bay Road, Newmarket, NH 03857, USA; 9 Office of Research and Development, Center for Environmental Measurement and Modeling, Atlantic Coastal Environmental Sciences Division, U.S. Environmental Protection Agency, Narragansett, Rhode Island, USA; 10 NOAA Fisheries, Northeast Fisheries Science Center, 166 Water Street, Woods Hole, MA 02543, USA; 11 Cape Cod Cooperative Extension, County of Barnstable, P.O. Box 367, Barnstable, MA 02630, USA; 12 Blue Oyster Environmental, LLC, 541 Poplar Street, Cambridge, MD 21613, USA; 13 Department of Agricultural and Applied Economics, Virginia Tech, Blacksburg, VA 24061, USA; 14 The Nature Conservancy Provide Food and Water Sustainably Team, 4245 Fairfax Drive, Suite 100, Arlington, VA 22203, USA; 15 Present address: Office of Aquaculture, NOAA Fisheries, SSMC3, 1315 East West Highway, Silver Spring, MD 20910, USA; 16 Ward Aquafarms, 51 N Falmouth Hwy, North Falmouth, MA 02556, USA; 17 Department of Biology and Department of Earth and Environment, Boston University, 5 Cummington Mall, Room 101, Boston, MA 02215, USA

**Keywords:** Denitrification, Oyster, Eutrophication, Nutrient management, Nitrogen

## Abstract

Nitrogen pollution is one of the primary threats to coastal water quality globally, and governmental regulations and marine policy are increasingly requiring nitrogen remediation in management programs. Traditional mitigation strategies (e.g., advanced wastewater treatment) are not always enough to meet reduction goals. Novel opportunities for additional nitrogen reduction are needed to develop a portfolio of long-term solutions. Increasingly, in situ nitrogen reduction practices are providing a complementary management approach to the traditional source control and treatment, including recognition of potential contributions of coastal bivalve shellfish. While policy interest in bivalves has focused primarily on nitrogen removal via biomass harvest, bivalves can also contribute to nitrogen removal by enhancing denitrification (the microbial driven process of bioavailable nitrogen transformation to di-nitrogen gas). Recent evidence suggests that nitrogen removed via enhanced denitrification may eclipse nitrogen removal through biomass harvest alone. With a few exceptions, bivalve-enhanced denitrification has yet to be incorporated into water quality policy. Here, we focus on oysters in considering how this issue may be addressed. We discuss policy options to support expansion of oyster-mediated denitrification, describe the practical considerations for incorporation into nitrogen management, and summarize the current state of the field in accounting for denitrification in oyster habitats. When considered against alternative nitrogen control strategies, we argue that enhanced denitrification associated with oysters should be included in a full suite of nitrogen removal strategies, but with the recognition that denitrification associated with oyster habitats will not alone solve our excess nitrogen loading problem.

## Introduction

Excess nitrogen loading is a leading cause of coastal ecosystem degradation globally (Breitburg et al. 2018). The symptoms of nitrogen pollution are well known and include eutrophication, harmful algal blooms, low oxygen conditions, and declines in biodiversity (Powers et al. 2005; Greening and Janicki 2006; Bricker et al. 2008; Breitburg et al. 2009; Turner et al. 2009). For over three decades, various strategies have been deployed to mitigate the negative impacts of excess nitrogen primarily in coastal waters across Europe, North America, Asia, and Australia (Boesch 2019). In the United States, the Clean Water Act (CWA) requires establishment of ambient water quality standards for rivers, lakes, and estuaries. Eutrophication due to excessive anthropogenic nutrient loads often results in a failure to achieve these standards. If this occurs, total maximum daily loads (TMDLs) are developed to establish the total amount of nitrogen that a waterbody can receive and still achieve the water quality standards. State and local governments are then responsible for developing nitrogen management plans to achieve nitrogen control targets in the TMDL (Copeland 2001; USEPA 2002). While there are success stories, nitrogen pollution is a recalcitrant problem, requiring both persistence and innovation to address (Fulweiler et al. 2012).

Nitrogen is particularly challenging to manage because it enters coastal water bodies through both point (e.g., sewage) and nonpoint (e.g., diffuse surface runoff from agricultural and urban lands and atmospheric deposition) sources and these require different management solutions. In the CWA, point sources (typically municipal and industrial wastewater treatment facilities) are permitted through the National Pollution Discharge Elimination System (NPDES; 33 U.S.C. §§1251–1387). Under nitrogen TMDLs, permits require wastewater treatment facilities to meet nitrogen effluent concentrations and load limitations (called wasteload allocations). These permit efforts have successfully reduced point source loading of nitrogen in several high-profile waterbodies such as Long Island Sound, Tampa Bay, and Chesapeake Bay (Greening et al. 2014; Varekamp et al. 2014; Ator et al. 2019). While initially developed for wastewater treatment facilities, in some cases, permitting with nitrogen effluent limitations has been extended to other sources such as municipal separate storm sewer systems (MS4s).

Many watersheds, however, are dominated by nonpoint source loads rather than point sources of nitrogen (Carpenter et al. 1998; Boesch 2019). Under a TMDL, nitrogen loads from such sources, called load allocation, must be reduced but often with few direct federal regulatory requirements. Nonpoint source nitrogen loading is arguably harder to manage because loads are expensive to identify, measure, and monitor. Regulating nonpoint sources is also more difficult due to the legal and practical difficulties of assigning nitrogen control responsibilities to nonpoint sources (Carpenter et al. 1998; Paerl et al. 2002; Minan 2005; Green et al. 2008). TMDL implementation plans typically rely on a variety of voluntary federal, state, and local programs to induce reductions in nitrogen nonpoint source loads. For example, federal and state programs provide landowners with financial assistance to adopt specific practices, such as cover crops, conservation tillage, and nutrient management plans, that reduce agricultural nitrogen runoff. Local governments may encourage reductions in urban nonpoint nitrogen through educational campaigns to reduce lawn application of fertilizer and financial inducements to adopt stormwater control measures such as rain gardens and rainwater harvesting (Houle et al. 2013; Gonzalez et al. 2016).

Given the challenges and limitations associated with managing nitrogen sources, in situ nitrogen reduction practices that remove nutrients from the water after they have entered the waterbody have also been growing in use (Malone 1984; Stephenson and Shabman 2017b). These practices typically function by enhancing a naturally-occurring biological community or habitat that is a net nitrogen sink in the environment. Some of these practices (e.g., floating wetlands, stream, and wetland restoration) are already included in existing nitrogen management plans in the United States (Phipps and Crumpton 1994; Craig et al. 2008; Mulbry et al. 2010; White and Cousins 2013).

One group of organisms that is being increasingly recognized for their contributions to in situ nitrogen reduction and potential relevance to nitrogen management is bivalve shellfish (Newell 1988; Lindahl et al. 2005). Bivalve shellfish remove nitrogen directly from the water column by assimilating filtered and ingested suspended particulates that contain nitrogen into tissue and shell biomass (Higgins et al. 2011; Rose et al. 2014; Bricker et al. 2020). Sequestered nitrogen may then either be retained in a stable habitat or removed from the system via harvest (Carmichael et al. 2012; Petersen et al. 2014; Rose et al. 2014). While implementation is currently geographically limited, enhancement and subsequent harvest of populations of eastern oysters (*Crassostrea virginica*) and hard clams (*Mercenaria mercenaria*) have been recently approved for inclusion in local (clams and oysters) and regional (oysters only) nitrogen management (Town of Mashpee Sewer Commission 2015; Cornwell et al. 2016). Enhancement (without harvest) of natural oyster biomass through reef restoration is currently being considered for inclusion as a nitrogen reduction practice by the Chesapeake Bay Program (Reichert-Nguyen 2018). Ingested particles that are not assimilated may be ejected as biodeposits and buried in the sediment, although data on this process are limited (Beseres Pollack et al. 2013; Kellogg et al. 2014; Lai et al. 2020).

A third potential pathway of nitrogen removal by shellfish is through enhancement of denitrification, the microbialdriven process of converting reactive (i.e., bioavailable) nitrogen to non-reactive di-nitrogen (N_2_) gas (e.g., Kellogg et al. 2013; Hoellein et al. 2014; Humphries et al. 2016; Smyth et al. 2016; Bilkovic et al. 2017; Zhu et al. 2019). Denitrification is unique, in that the nitrogen is no longer bioavailable and is removed from the immediate ecosystem where water quality problems can occur. Bivalve shellfish may enhance local denitrification rates by concentrating organic matter in underlying sediments, where the carbon and nitrogen from biodeposits, and its eventual decomposition, may provide reactants and conditions needed to support denitrification and lead to increases in nitrogen removal even in eutrophic areas (Zhu et al. 2019). Microbial communities in the anoxic guts of shellfish and on their shells may further contribute to denitrification (Caffrey et al. 2016; Arfken et al. 2017; Ray et al. 2019).

Oyster-mediated denitrification has been measured in a variety of coastal and estuarine ecosystems. In some locations, substantial enhancement of denitrification has been documented over bare sediment controls (e.g., Kellogg et al. 2013; Humphries et al. 2016), but this has not been observed in all locations and/or at all times within a single location (e.g., Higgins et al. 2011, Westbrook et al. 2019). A recent metaanalysis, however, examined the available data on directly measured sediment denitrification under oyster reefs and aquaculture farms (Ray and Fulweiler 2021). They report that oysters have a strong positive effect on denitrification in both scenarios. While the effect of oysters on denitrification was higher in reef habitats compared to aquaculture habitats, there was no statistical difference between the two, suggesting that both habitats increase nitrogen removal equally (Ray and Fulweiler 2021).

Here, we describe the opportunities and challenges of incorporating bivalve-mediated denitrification into existing nitrogen management programs. The inclusion of oyster-mediated denitrification in nitrogen management is also currently under review by the multi-state Chesapeake Bay Program (Reichert-Nguyen 2018). We focus on oysters because of their wide-spread and growing role in both aquaculture and reef restoration and consider how including oyster driven denitrification in nitrogen management plans may impact both of these management areas. We discuss policy options to support expansion of oyster-mediated denitrification, describe the practical considerations for incorporation into nitrogen management, and summarize the current state of the field in accounting for denitrification in oyster habitats.

## Opportunities to Integrate Oyster-Mediated Denitrification into Nitrogen Management Programs

**Fig. 1.**
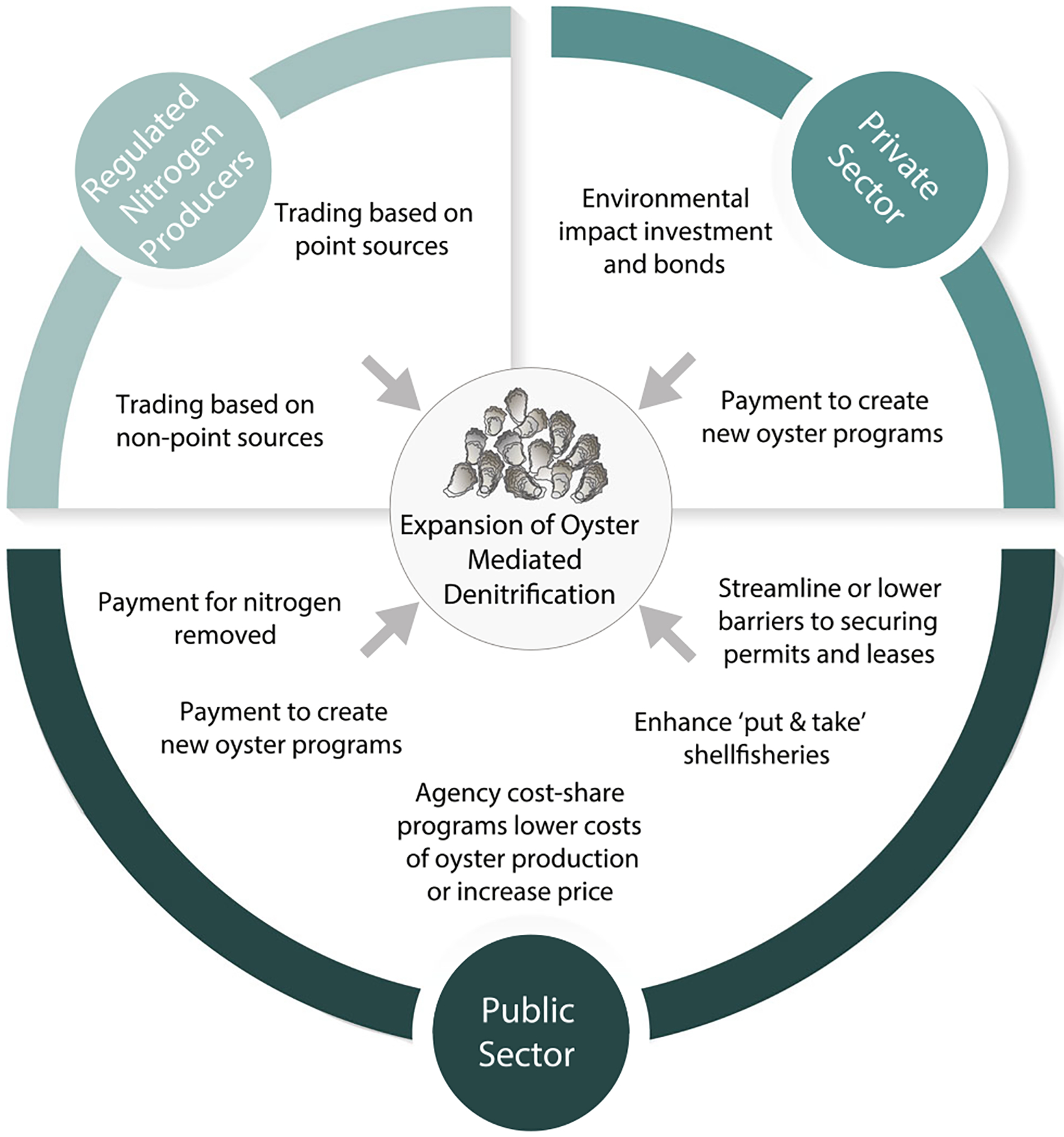
Policy options to support expansion of oyster-mediated denitrification. Opportunities are categorized based on sector, and include both financial and nonfinancial mechanisms that could increase implementation of restoration and aquaculture practices. Oyster graphic by Tracey Saxby via https://ian.umces.edu/imagelibrary/displayimage-4336.html

Expansion of oyster-mediated denitrification via increased aquaculture and restoration can be encouraged with payment and other support for these services. A variety of policy options exist to support the expansion of oyster-mediated denitrification by the public and private sectors, and several options have been piloted and used to aid water quality management efforts (Fig. 1).

State and local governments’ efforts to mitigate nitrogen input from unregulated nonpoint sources offer a diverse range of policy options and opportunities to support and expand implementation of oyster-mediated denitrification enhancement practices (Fig. 1—public sector). For most nonpoint source programs, financial assistance is typically provided based on the installation or maintenance of a practice or activity that is intended to remove nitrogen. Federal and state “cost-share” programs pay a portion of the costs to install practices such as cover crops, stream buffers, and livestock exclusions from streams. A variety of practice-based financial assistance programs can support oyster-based denitrification, including subsidies for oyster aquaculture gear, oyster seed costs, and payments for enhanced shelling of a barren seafloor (Bosch et al. 2010). If these efforts are successful in increasing oyster numbers and denitrification, progress can be claimed within a TMDL that recognizes denitrification as an acceptable water quality control option.

Government agencies responsible for TMDL implementation also have a number of other options to expand oyster production to enhance nitrogen removal. These actions could indirectly lower the price of inputs needed for oyster production or enhance oyster prices, thus facilitating additional investment in oyster production. State and/or local agencies could streamline and lower barriers to securing permits and leases to expand oyster production, an approach being piloted by Falmouth, MA, through the siting of aquaculture development zones (Town of Falmouth 2017). Another alternative to increase production is through enhancements to put-and-take fisheries, currently being implemented in Mashpee, MA (Town of Mashpee Sewer Commission 2015). Local governments may alter zoning requirements or property taxes to support oyster infrastructure (e.g., hatchery production and oyster processing facilities). Local and state programs could also create certification programs that promote the environmental stewardship provided by local oyster production (Kuminoff et al. 2008). These actions could be part of a TMDL implementation plan, and local governments could claim progress toward reducing nitrogen loads to a targeted waterbody.

Oyster-mediated denitrification can be supported directly through implementation of payment for ecosystem services (PES) programs, whereby producers of ecosystem services (e.g., nitrogen removal) are compensated by beneficiaries to continue providing the services (Farley and Costanza 2010). In PES programs for water quality, compensation is paid based on the quantity of pollutants removed per unit of time (e.g., USD kg^−1^). A variety of nitrogen reduction practices— including both traditional infrastructure and nature-based solutions—have been implemented or proposed, from in situ practices such as stream or wetland restoration to sewering. Beneficiaries could be public agencies seeking to achieve public water quality goals or regulated entities that need to offset nitrogen discharge. In the latter case, nitrogen trading programs have been proposed as a mechanism to enable regulated nitrogen sources to meet nitrogen reduction goals by paying other entities to help meet their nitrogen load requirements (WLA) (Willamette Partnership and World Resources Institute 2015). Regulated sources may be interested in trading as a way to offset unavoidable growth or as a way to reduce compliance costs. These other entities (sellers) could be other point sources who discharge nitrogen below their permit levels and thus have “excess” nitrogen available for trading. The excess nitrogen control created by the seller must be quantified on a mass load basis (kg) and defined in both temporal and spatial dimensions, often called a credit. Alternatively, the regulated source may be allowed to purchase nitrogen credits from unregulated nonpoint sources, typically agriculture sources (sometimes called “point–nonpoint” trading). Nitrogen credits, however, could also be generated and sold by entities who actively invest to increase in situ nitrogen removal processes (Stephenson and Shabman 2017b). This could include nutrient sequestration or practices that enhance oyster-mediated denitrification (Fig. 1—nitrogen producers).

Trades involving nonpoint source reduction or in situ nitrogen removal approaches have been limited to date (Stephenson and Shabman 2017c). Only two active nitrogen trading programs in the United States currently allow nutrient credit trades involving oysters, Virginia’s Nutrient Credit Exchange and Maryland’s Nutrient Trading Program. Demand for nonpoint source credits within the Virginia program is currently low (Stephenson and Shabman 2017a). Maryland’s program has only recently been implemented, and trading thus far has been limited (Maryland Department of the Environment 2020). The low volume of trading is partly due to limited demand from permitted sources. Federal permitting programs are, by design, intended to maximize point source reductions so regulatory programs act to limit the ability of permitted sources to achieve compliance using thirdparty sources (Stephenson and Shabman 2017c). Nitrogen trading programs involving regulated nonpoint sources, such as municipal separate storm sewer (MS4) programs, may be a promising option for future trades involving oyster-mediated denitrification enhancement. However, to be attractive to any potential buyer, the nitrogen removal costs ($ lb^−1^ year^−1^) of in situ projects would need to be competitive with those incurred by point and nonpoint sources that currently offer credits. Within existing point source trading programs, the price of nitrogen credits is typically less than $10 lb^−1^ year^−1^ (CTDEEP 2018; Virginia Nutrient Credit Exchange Association 2020). Recent demonstration trades involving nitrogen removal through assimilation into oyster tissue involved one-time payments to two growers of $50–400 lb^−1^ year^−1^ (Wheeler 2020).

The private sector could also provide financial support for expansion of oyster denitrification (Fig. 1—private sector). For “impact investors”—those seeking financial, social, and environmental returns on investments—valuing oyster-mediated denitrification allows for evaluation of the magnitude of environmental or social impact opportunity relative to other options (e.g., investment in other nitrogen reduction approaches) and could inspire further investment (O’Shea et al. 2019). In some cases, the financial support may be voluntary donation. In others, investors may provide financing only under the expectation that another party will repay once services are provided. For example, “environmental impact bonds” have been proposed as an option for private investors to provide funds to local or state governments for implementation of nitrogen reduction practices (e.g., oyster restoration), with a return on their investment only upon a successful outcome.

## Consideration for Establishing a Policy Framework

### Timing and Location of Nitrogen Reduction Delivery

As an in situ nitrogen removal practice, the physical location of oysters in the impaired waterbody may lead to a suite of nitrogen removal benefits that differ from land-based alternatives (Table 1). Water quality equivalence refers to the relating of different nitrogen removal practices to achieving the same desired ambient water quality response (e.g., increased dissolved oxygen) (Stephenson and Shabman 2017b). Since oyster-associated nitrogen removal via denitrification occurs directly in the water, nitrogen removal is not delayed by nitrogen transport and attenuation within the watershed itself (Keller et al. 2014). This provides the benefit of creating more immediate positive impacts in situations where upstream nitrogen reduction efforts may be delayed by social/political inertia or lag times in source control associated with nitrogen transport times (Meals et al. 2010). For example, in groundwater fed systems like Cape Cod, MA, or Long Island, NY, USA, the delay in water and nutrient transport can result in decades-long travel times for nutrients from upstream sources to estuarine waters. Thus, historic or legacy loads will continue to affect an impaired waterbody for decades or longer after upstream source controls are implemented (Van Meter et al. 2016). At the same time, forgoing upstream investments in source control in favor of in situ approaches, implies allowing for continuing damages nitrogen may cause in transport, say in intercepting ponds. Therefore, while in situ approaches may complement source control efforts, and potentially enhance the cost-efficiency, and immediacy of pollution control efforts, they are not substitutes for one another.

### Ensuring Effectiveness of Payments for Increasing Implementation of Nitrogen Reduction Practices

Since oyster-mediated denitrification also provides the opportunity to achieve nitrogen reduction without physical removal of the organisms from the local environment (as opposed to nitrogen removal via biomass extraction), integrating bivalve-mediated denitrification into nitrogen management plans could have major impacts on a variety of conservation and resource management plans. For restoration projects, payments for nitrogen removal services would provide a continual, quantifiable benefit that might motivate additional reef restoration activities and lead to an increase in restored acreage in sanctuaries or other areas protected from harvest; similar impacts have been proposed from valuing other services (Grabowski et al. 2012). Currently, most restoration activities are funded via public or private grants. However, the rate at which oyster habitat is being lost far exceeds the capacity of current public funding for restoration (Hernandez et al. 2018). Payments for nitrogen removal services add value since reefs would be considered a mitigation strategy, if sources of funding can be identified. This valuation may encourage local community “buy-in” and create further incentives for private entities to invest in restoration of degraded systems.

To be effective, the financial and nonfinancial incentive systems acknowledging oyster-mediated denitrification must increase the number of oysters placed in coastal waters through both aquaculture and restoration over what would have occurred in an absence of the program. Additionality is the incremental level of nitrogen removal achieved from what would have occurred in an absence of active management policy efforts. The result would be payments for services that would have been provided anyway, without payment, through the course of normal business operations (Flood 2019). Additionality may be particularly challenging within the context of shellfish aquaculture where positive financial returns are already being achieved from the sale of oysters alone (Table 1). In such a setting, existing oyster producers are providing nitrogen removal services for free. One policy challenge is to determine how and when to start “counting” new or additional production. Establishing such a “baseline” is empirically and practically challenging in this context (Stephenson and Shabman 2017b). Establishing baselines must address equity issues since new entrants or marginal producers could be subsidized at the expense of existing producers that would operate profitably without payments. Nonadditionality also raises potentially adverse water quality outcomes. Within a trading context, a nitrogen discharger could purchase nitrogen credits from an existing oyster operation in order to increase nitrogen loads without any corresponding increase in denitrification.

The impact of any payment of nitrogen removal services program, including credit trading programs, on aquaculture growth will also depend partly on the value of the credit or payments. Economic modeling has suggested that higher credit prices will facilitate growth in the aquaculture industry (Weber et al. 2019). The effectiveness of payments in achieving nutrient reduction may be limited by other constraints farmers face, such as lease area, available working waterfront space, and market for their product.

### Alteration of Farming Practices

The type of financial incentive system can alter the size and characteristics of oyster production. For example, input subsidies can skew oyster investments toward specific types of inputs or production methods. PES systems that relate oyster size to higher levels of oyster processing may lead to changes in the size composition of marketed oysters (Taylor et al. 2019). For example, if denitrification is impacted by stocking density, harvest size, ploidy, or farming strategy (e.g., off-bottom vs. bottom cultivation), this could lead to the use of new techniques or possible production of larger oysters. Increased oyster production may create the need for the oyster aquaculture industry to develop new or differentiated markets for their product and the equipment needed to support them, especially if nitrogen reduction is influenced by cultivation practices. In this sense, payments for nitrogen reduction services may provide incentives for industry investment in long-term sustainability of the environment, fishery, and community. For example, oyster production in the Northeastern United States is almost entirely (95+%) for the half-shell market sold by the piece, though there are alternative markets for shucked or processed product (The Hale Group 2016). These alternative markets may have greater capacity to handle additional growth in volume, but local infrastructure to support farming strategies that maximize denitrification may not currently exist throughout the region. Additional revenue from nitrogen reduction payments could make that avenue more attractive to businesses and investors.

### Ancillary Benefits Provided by Oysters

Enhancement of oyster populations, whether through aquaculture or reef restoration practices, would also provide additional ecosystem services beyond nitrogen reduction. Ancillary benefits in addition to nitrogen reduction are often a consideration in developing TMDL implementation plans (Table 1). The filtering of water by oysters can increase water clarity and improve conditions for species such as seagrasses (Newell and Koch 2004; Wall et al. 2008). Some evidence suggests that oyster feeding and growth might also sequester carbon (Fodrie et al. 2017). Oysters also directly provide habitat for other organisms as they grow via reef formation. Support of commercially and recreationally important finfish stocks through habitat provisioning may lead to additional ecosystem services (Gilby et al. 2018). Colonization of oyster reefs or aquaculture gear by other suspension feeding organisms may further enhance water clarity improvements and support additional denitrification enhancement (Kellogg et al. 2018). Oyster reefs have also been shown to provide shoreline protection through wave attenuation and shoreline stabilization (Meyer et al. 1997; La Peyre et al. 2014). Oysters in reefs (Peters et al. 2017) and aquaculture settings (Varney et al. 2018) can contribute to stock enhancement through larval output, as long as diploid oysters (i.e., capable of reproduction) are used. Although issues concerning mixture of gene pools may need to be addressed (Jaris et al. 2019), these studies highlight the potential for oyster populations to be self-sustaining and require less maintenance than other nitrogen removal tools. Further, an increase in aquaculture production of oysters could contribute shell materials useful for restoration activities (reef or bed enhancement) and buffer against ocean acidification (Filgueira et al. 2015). Importantly, any of these “ancillary” benefits may actually be the driver of reef restoration programs. Valuing nitrogen removal via denitrification would add benefits to these projects and similarly aid their growth even outside a TMDL framework.

## Siting Considerations

The nature of in situ oyster-mediated denitrification also means that some sites may not be suitable for this approach (Table 1). Environmental considerations affecting siting include factors such as temperature, salinity, water quality, currents, flushing times, and availability of sufficient food quality and quantity to support oyster growth. Social considerations may lead to constraints on production inputs, such as opposition from coastal residents and competing spatial uses of coastal or estuarine waters, such as recreational boating and commercial fishing (Dalton et al. 2017). Interactions among stakeholders and resulting regulations, combined with noted spatial differences, may affect implementation of some of the incentivization mechanisms we have described.

Availability of space for cultivation or restoration may present additional challenges (Beckensteiner et al. 2020). A potential constraint that combines social and environmental considerations is the presence of high bacterial abundance in a location that is also experiencing nitrogen-related water quality impairments. Harvest of shellfish from waters with high bacterial abundance is prohibited, precluding aquaculture activities for public consumption. Oyster reef restoration in closed or prohibited waters has been viewed by some states as an “attractive nuisance” due to public health concerns surrounding the potential for illegal harvest, personal consumption, or even sale into the commercial food supply. A lack of adequate resources for enforcement to prevent illegal harvest precludes oyster restoration projects in closed waters in US states such as New Jersey and Massachusetts (Holley et al. 2018). In areas where environmentally and socially suitable areas for aquaculture and restoration do not coincide with watershed impacts to water quality, credit trading programs may facilitate the development of “hotspots” where nutrient removal benefits are realized in areas spatially removed from the impacts. Regulatory programs governing nutrient credit trading must explicitly address the spatial distribution of the benefits and impacts to ensure the expected water quality benefits are realized.

Siting challenges may be addressed through the use of a growing number of GIS-based mapping tools to aid in the identification of appropriate sites that minimize user conflict and have no bacteria-based water quality restrictions (Wickliffe et al. 2019). The incorporation of local monitoring data relevant to oyster growth into these mapping tools would further improve siting of oyster-mediated denitrification enhancement practices (Bricker et al. 2016).

## Quantification of Nitrogen Removal

Denitrification is a nitrogen removal pathway for many common nitrogen reduction best management practices (see Table 1), and quantification of this removal pathway must be addressed in any nitrogen management program. If a site is environmentally suitable and socially acceptable for oyster installation, a further challenge will be to properly estimate the impacts of oysters on nitrogen removal for achieving water quality goals and evaluating effects of management alternatives. A related policy challenge is the level of certainty in the magnitude of nitrogen removal provided, given observed variability in denitrification enhancement across space and time. There are generally three approaches to quantifying nitrogen removal from oyster habitats: direct measurement, measurement of indirect proxies, and ecological modeling. In nitrogen management programs, the vast majority of nitrogen reduction strategies utilize modeled approaches (Table 1).

### Direct Measurement

A recent review has identified best practices for documenting oyster-mediated denitrification enhancement associated with either oyster aquaculture or restoration practices (Ray et al. in revision). A set of recent recommendations from the Chesapeake Bay Program included site-specific measurements of denitrification to determine local rates due to variability derived from the complex feedbacks that occur between bivalves, the water column, and the reef microbial community (Reichert-Nguyen 2018). Oysters interact with the water column by impacting surrounding hydrodynamic conditions, ultimately affecting particle transport and leading to changes in food availability and particle concentrations. Individual oyster filtration rates are a function of oyster size, age, particle availability, temperature, and salinity (Ehrich and Harris 2015). These processes ultimately affect rates of biodeposit production, and the biodeposits themselves are subject to resuspension and transport before they become available for either burial, N-removal via denitrification, or ammonification that results in a recycling of N back to the water column (Testa et al. 2015).

The rates of nitrogen removal will be dependent on the local environment experienced by a given oyster, especially in terms of average current velocities and phytoplankton and suspended sediment concentrations, as well as the ways that ambient salinity and temperature impact a variety of physiological factors. Physical configuration of the oysters in a reef, and variations in aquaculture cultivation practices, may also impact nitrogen removal. For example, for off-bottom aquaculture, bottom sediment quality and the presence of nitrifying and denitrifying microbes are important. Sites with poor sediment conditions (e.g., high hydrogen sulfide concentrations) will result in minimal or no denitrification (Higgins et al. 2013). Changes in oyster density would also impact nitrogen removal and thus need to be monitored. An excess of oysters could result in overabundance of biodeposits resulting in poor bottom sediment conditions and thus decreased rates of nitrification/denitrification. Although it is important to highlight that as of now, when taken together, the current available data demonstrates that sediments from oyster habitats have higher rates of nitrogen removal via denitrification than bare sediments (Ray and Fulweiler 2021). While oysters may form self-sustaining populations that are resilient to disasters such as hurricanes and droughts that may render other management strategies ineffective, the isolated or synergistic impacts of disease, predator outbreaks, and other environmental changes may limit population growth and even lead to catastrophic population loss (Garland and Kimbro 2015).

### Proxies for Denitrification Enhancement

If widespread use of shellfish-induced denitrification is to occur within water quality management programs, straightforward and low-cost options are needed to quantify the nitrogen removal services from enhanced denitrification (Groffman et al. 2006). Some evidence suggests that proxies could be used to quantify denitrification enhancement. Sisson et al. (2011) observed a relationship between denitrification rates and oyster biomass in experiments conducted at a reef restoration site in Chesapeake Bay. Others (Piehler and Smyth 2011; Hoellein and Zarnoch 2014; Zhu et al. 2019) have observed positive relationships among sediment organic content, sediment oxygen demand, and denitrification rates. A general impact of oyster presence on denitrification rates could also be determined, mirroring an approach that has been used to estimate impacts of vegetation on denitrification rates (Alldred and Baines 2016). Monitoring of oyster biomass or sediment properties could provide assurance to nitrogen management programs that locally established denitrification rates continue to be relevant at a much lower cost than sustaining a time series of denitrification measurements. Continued research into low-cost predictors of denitrification would increase the likelihood that oyster-mediated denitrification enhancement practices would be used in nitrogen management programs.

### Model Development

Active use of shellfish-related denitrification as a water quality management option will be greatly increased with the development of sufficiently acceptable models to quantify the levels of nitrogen removal through denitrification. Predicting denitrification in estuarine settings is not a new modeling challenge. A process-based model for simulating sediment biogeochemistry processes in coastal ecosystems was initially developed by Di Toro (2001) as the “sediment flux model” (SFM). This forms the basis for simulating benthic–pelagic coupling in the Chesapeake Bay Eutrophication Model (Cerco and Noel 2004), as well as models for Long Island Sound (HydroQual 1991), Massachusetts Bay (HydroQual and Normandeau Associates 1995), and several other coastal systems. Cerco (2015) adapted these formulations by including bivalves to predict nutrient removal by oysters in the Great Wicomico River sub-estuary. The SFM also forms the basis for a modeling effort by Testa et al. (2015) to simulate biogeochemistry around floating oyster farm aquaculture in combination with hydrodynamic processes that act upon biodeposits that are ultimately the organic material that fuels denitrification and burial of nitrogen in and around oysters. Harris et al. (2019) have combined a 2-D hydrodynamic particle tracking model with model formulations that simulate filtration rates (Ehrich and Harris 2015), biodeposition, and the SFM to consider how reef morphology, oyster size and density, and environmental conditions such as current velocity, chlorophyll-a concentrations, temperature, and salinity combine to influence nitrogen cycling and removal. These models have the capacity to investigate how multiple factors combine to affect removal of phytoplankton from the water column and facilitation of burial, recycling, or denitrification using SFM. The challenge of using these models is their high data requirements for parameterization, but a hope for future application is that general relationships may be used to make predictions about oysters related to nitrogen removal.

Reduced complexity models that are designed for end-user input such as the farm aquaculture resource management (FARM) model also estimate nitrogen removal (Rose et al. 2015; Bricker et al. 2018). While applications of FARM have generally been restricted to computing nitrogen removal through bioextraction (i.e., sequestration in tissues and shells with subsequent harvest), Bricker et al. (2020) combined FARM model estimates of bioextractive removal with published local measurements of oyster-mediated denitrification to provide a more holistic estimate of oyster-associated nitrogen removal in Great Bay; NH. Kellogg et al. (2018) applied a reduced complexity box model rooted in site-specific observations to the tributary-scale oyster restoration in Harris Creek, MD. The model projected that restored oysters are now removing over 200% of watershed nitrogen inputs. Denitrification was responsible for the largest fraction of these removals (73%), followed by sequestration in shell (13%), tissue (10%), and burial (3%). The model is now available online for stakeholders to estimate nitrogen removals as a function of restored area, oyster density, and oyster size (Kellogg and Brush 2018). These models do not have the fine spatial and mechanistic detail of the SFM or particle-tracking hydrodynamics, but nevertheless reproduce the observations, are easily parameterized, can still be tailored to site-specific locations, and can be served online. Both types of models (finely resolved and reduced complexity) have the potential to inform nutrient management programs that incorporate oyster-mediated denitrification. Additional research is needed to compare predictions produced by these different types of models, to further hone them to site-specific applications, and to build consensus on appropriate modeling tools.

Modeling and quantification efforts should be evaluated within the policy context of ambient water quality management. Water quality managers may consider the level of certainty and confidence in nitrogen removal practices to generate specific levels of nitrogen control. In the policy context, uncertainty of model estimates of oyster-mediated denitrification enhancement should be evaluated against the uncertainties and costs (Rose et al. 2015) associated with other nitrogen removal practices to achieve ambient water quality goals, in particular nonpoint source load reductions (Table 1). Nonpoint source management efforts within the TMDL program rely almost exclusively on modeled estimates of the effectiveness of nonpoint source management actions (Table 1; e.g., best management practices like bioretention, cover crops, riparian buffers). Beyond permitted sources, monitoring within a TMDL occurs at the ambient level to track overall progress toward achieving water quality standards but not at the level of tracking the nitrogen removal outcomes of individual nonpoint source management actions.

Water quality managers commonly accept and rely on average removal efficiencies for nonpoint source practices that have considerable observed ranges in control effectiveness (Liu et al. 2017; Stephenson et al. 2018; Aguilar Marcus and Dymond Randel 2019). For example, most urban and agricultural nonpoint source best management practices (BMPs) have multiple nitrogen removal processes and removal pathways. The pathways are not all thoroughly characterized in the literature, and expert judgment is often used in place of models to determine removal effectiveness rates of BMP types (Stephenson et al. 2018). Yet, these practices form the backbone of most nonpoint source control efforts. While oyster denitrification is subject to complex and site-specific conditions, the level of uncertainty in nonpoint source effectiveness may be considered as useful references when evaluating and quantifying in situ removal options.

## Conclusions

This summary shows the potential of, and challenges to, inclusion of oyster denitrification in nitrogen management programs. In many respects, oyster denitrification is not unique to water quality management, as denitrification is an important nitrogen reduction pathway for many land based nonpoint source practices (Table 1). Verification of oyster-mediated denitrification may be facilitated by being able to quantify the oyster biomass responsible for enhancing nitrogen processing. Development of models to estimate denitrification enhancement associated with oyster habitats may yield more widespread implementation by nitrogen management programs.

Oyster-mediated denitrification enhancement is emerging as one of several nitrogen reduction tools employed by resource managers, and the discussion here illustrates its potential to contribute to coastal and estuarine nitrogen management. Similar arguments could be made for consideration of denitrification enhancement by other organisms, such as clams, mussels, and wetland plants (Nizzoli et al. 2006; Bastviken et al. 2007; Alldred and Baines 2016; Bilkovic et al. 2017; Zhu et al. 2019). Recognizing and valuing this additional nitrogen reduction service may encourage the application of in situ practices and expand their contributions to overall nutrient management. While the challenges we have described here for oyster-mediated denitrification enhancement will likely exist for other species, the benefits of employing these methods and markets to support them likely exist as well. Beginning to account for this ecosystem service will encourage advances in both management and research.

Given the noted benefits and challenges associated with integrating oyster-mediated denitrification into nitrogen reduction plans, an adaptive management approach will be critical for successfully implementing new practices and integrating them into the broader approach to nitrogen management. Adaptive management, which is an iterative process by which management actions are modified in response to progress on achieving objectives, is thought to be useful for dealing with the inherent uncertainty in the management of complex systems (Eberhard et al. 2009). Adaptive management can be useful in areas of active research, where new findings can influence implementation of management programs. This is true for many novel management tools. For example, the Chesapeake Bay Program recognized the potential for stream restoration projects to result in increased denitrification rates, considered the uncertainty associated with estimates of those denitrification rates, and approved a best management practice that credits denitrification enhancement associated with stream restoration (Berg et al. 2013). The expert panel for this best management practice noted that available data did not allow a “perfect estimate” of nitrogen removal, but also recognized the benefits of this approach and decided to support its use while closely monitoring results. The Chesapeake Bay Program noted that although nitrogen removal associated with stream denitrification was calculated using a single equation, adaptive management processes allow variability in this rate to be re-considered as more science becomes available, and in fact, this BMP was re-evaluated and the credited rates of denitrification enhancement were updated several years later (Altland et al. 2020).

In summary, it is clear that more tools are needed to reduce excess anthropogenic nitrogen in estuaries. For bivalve shellfish such as oysters, we should consider the full potential for nitrogen reduction, including denitrification, and methods to incentivize these in situ practices. Current science is able to directly measure microbially-mediated denitrification and the scientists are actively developing more robust tools to estimate these rates with both empirical relationships and mechanistic models. Finally, the adaptive management framework employed by coastal and estuarine resource managers is sufficiently sophisticated to integrate this nuance into both restoration and aquaculture contexts, with a diversity of potential implementation frameworks.

## Figures and Tables

**Table 1.** Comparison of oyster denitrification enhancement to other established nitrogen reduction best management practices. Information on other practices compiled fromAdler et al. (2014), Bahr et al. (2012), Bott et al. (2015), Coale et al. (2016), Cornwell et al. (2016), and Staver et al. (2017)

Nitrogen reduction practice	N reduction pathways	N source typically targeted	Timing of N reduction delivery	N reduction quantification	What is verified	Ancillary benefits	Siting considerations	Sources of uncertainty
Oyster denitrification enhancement	Denitrification	Any (ambient water)	Immediate, depends on sediment conditions	To be determined	Population	Increased water clarity, habitat creation, shoreline protection, carbon sequestration, stock enhancement, buffering against ocean acidification	Oyster growth conditions, coastal use conflicts, water quality constraints	Variability in N removal with oyster abundance, biodeposition production/fate; potential additionality issue with aquaculture
Shellfish nitrogen assimilation	Capture/removal	Any (ambient water)	Immediate, relatively constant	Measured	Harvest	Phosphorus reduction; increased water clarity, habitat creation	Oyster growth conditions, coastal use conflicts, water quality constraints	Sediment nutrient release from bivalve biodeposition; loss of benthic biota if organic matter loading is excessive; potential additionality issue with aquaculture
Algal turf scrubber	Capture/removal	Any (ambient water)	Immediate, relatively constant	Measured	Harvest	Phosphorus and sediment reduction, dissolved oxygen production	Potential	wildlife/habitat impacts
Crop fertilizer reductions	Source reduction	Agricultural (unregulated nonpoint)	Variable, depends on transport, weather	Estimated	Nitrogen management plans	Phosphorus reduction		Reduced crop yield; verification of implementation difficult to document
Cover crops	Storage	Agricultural (unregulated nonpoint)	Variable, depends on transport, weather	Estimated	Practice installation	Phosphorus and sediment reduction	Less effective on steep slopes, stony soils, and wet conditions	Removal dependent on early planting
Crop to forest conversion	Storage; denitrification	Agricultural (unregulated nonpoint)	Delayed and variable	Estimated	Practice installation	Habitat creation, phosphorus and sediment reduction		
Bioretention areas	Storage; denitrification	Stormwater (regulated and unregulated nonpoint)	Variable, depends on transport, weather	Estimated	Practice installation	Phosphorus and sediment reduction	Limited space availability for urban retrofits	Regular maintenance required to ensure continued function
Wet ponds	Storage; denitrification	Stormwater (regulated and unregulated nonpoint)	Variable, depends on transport, weather	Estimated	Practice installation	Phosphorus and sediment reduction	Limited space availability for urban retrofits	Regular maintenance required to ensure continued function
Stormwater treatment wetlands	Storage; denitrification	Stormwater (regulated and unregulated nonpoint)	Variable, depends on transport, weather	Estimated	Practice installation	Phosphorus and sediment reduction	Limited space availability for urban retrofits	Regular maintenance required to ensure continued function
Bioreactors	Denitrification	Groundwater (unregulated nonpoint)	Immediate, relatively constant	Estimated/measured	Practice installation		Must intersect entire groundwater plume	Maintenance required; changed oxidation state may result in release of new dissolved contaminants
Wastewater treatment upgrades	Denitrification	Wastewater (regulated point)	Immediate, relatively constant	Measured	Sampling/measurement			
Advanced septic systems	Storage; denitrification	Septic (regulated and unregulated nonpoint)	Delay in transport	Estimated	Practice installation	Sediment reduction; pathogen removal	Soil texture affects performance	Regular maintenance required to ensure continued function; alkalinity control necessary to function effectively
Septic to sewer conversion	Denitrification	Septic (regulated and unregulated nonpoint)	Delay in transport	Estimated	Practice installation			
